# Individual-patient prediction of meningioma malignancy and survival using the Surveillance, Epidemiology, and End Results database

**DOI:** 10.1038/s41746-020-0219-5

**Published:** 2020-01-30

**Authors:** Jeremy T. Moreau, Todd C. Hankinson, Sylvain Baillet, Roy W. R. Dudley

**Affiliations:** 1 McConnell Brain Imaging Centre, Department of Neurology and Neurosurgery, Montreal Neurological Institute, McGill University, Montreal, QC Canada; 2 Department of Pediatric Surgery, Division of Neurosurgery, Montreal Children’s Hospital, Montreal, QC Canada; 3 Department of Pediatric Neurosurgery, Children’s Hospital Colorado, University of Colorado Anschutz Medical Campus, Aurora, CO USA; 4 Morgan Adams Foundation Pediatric Brain Tumor Research Program, Aurora, CO USA

**Keywords:** Cancer epidemiology, CNS cancer, CNS cancer, CNS cancer, Predictive markers

## Abstract

Meningiomas are known to have relatively lower aggressiveness and better outcomes than other central nervous system (CNS) tumors. However, there is considerable overlap between clinical and radiological features characterizing benign, atypical, and malignant tumors. In this study, we developed methods and a practical app designed to assist with the diagnosis and prognosis of meningiomas. Statistical learning models were trained and validated on 62,844 patients from the Surveillance, Epidemiology, and End Results database. We used balanced logistic regression-random forest ensemble classifiers and proportional hazards models to learn multivariate patterns of association between malignancy, survival, and a series of basic clinical variables—such as tumor size, location, and surgical procedure. We demonstrate that our models are capable of predicting meaningful individual-specific clinical outcome variables and show good generalizability across 16 SEER registries. A free smartphone and web application is provided for readers to access and test the predictive models (www.meningioma.app). Future model improvements and prospective replication will be necessary to demonstrate true clinical utility. Rather than being used in isolation, we expect that the proposed models will be integrated into larger and more comprehensive models that integrate imaging and molecular biomarkers. Whether for meningiomas or other tumors of the CNS, the power of these methods to make individual-patient predictions could lead to improved diagnosis, patient counseling, and outcomes.

## Introduction

Meningiomas are the most common primary central nervous system (CNS) tumor, with an incidence of 8.14 per 100,000 population.^1^ They typically present with gradual onset of symptoms in the later decades of life and have generally favorable outcomes relative to other CNS tumors.^2^ However, there is a great deal of variability in both aggressiveness and outcomes.^3^ The decision to opt for a “watch-and-wait” approach is made in around half of patients,^4^ but the process leading to this decision making remains challenging and often relies on simple heuristics, which may or may not be based on up to date evidence. The ability to precisely predict meningioma malignancy and survival beyond this standard would therefore be of clinical significance.

Many efforts to date in applying machine learning methods to the detection and grading of meningiomas have focussed on magnetic resonance imaging (MRI) imaging characteristics in small samples of patients. In this study, we develop and validate new predictive models using a set of basic clinical variables available in the Surveillance, Epidemiology, and End Results (SEER) database to predict meningioma malignancy and survival after specific treatments. The models are trained and tested on 62,844 patients included in SEER, an authoritative population-based cancer dataset with ~28% coverage of the US population.^5^ A new smartphone and web app was also developed to accompany this manuscript (www.meningioma.app).

Rather than being used in isolation, we expect that the proposed models will be integrated into larger and more comprehensive models that will integrate imaging and molecular biomarkers. The source code of the meningioma.app also provides an easy entry-point for future investigators to translate predictive models for broader dissemination.

## Results

### Malignancy

**Fig. 1:**
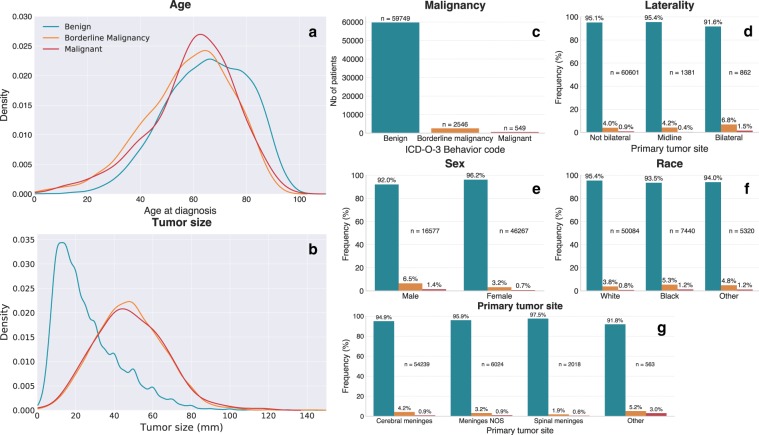
Descriptive statistics for the malignancy outcome variable. Kernel density plots illustrate the distribution of benign, borderline malignant, and malignant meningiomas according to age at diagnosis (**a**) and tumor size (**b**). These kernel density plots are conceptually equivalent to histograms, but illustrate density (i.e., relative number of patients) as a continuous function of age/tumor size. Total number of meningiomas by WHO ICD-O-3 behavior codes are shown in **c**. Absolute numbers and percentages of patients with benign, borderline malignant, and malignant meningiomas by subgroup are shown for laterality (**d**), sex (**e**), race (**f**), and primary tumor site (**g**).

Descriptive univariate statistical results of features initially included in the Balanced Logistic Regression-Random Forests (BLR-RF) model are presented in Fig. 1. No inferential statistical tests were performed as the goal of these exploratory analyses was solely to identify features with potential discriminatory value in relation to the outcome variables. Younger patients, and patients below the age of 20 especially, had relatively more malignant and borderline malignant meningiomas than older patients (Fig. 1a). Conversely, the relative prevalence of benign meningiomas was higher in older patients. In absolute numbers, however, benign meningiomas were much more frequent than borderline malignant or malignant meningiomas (Fig. 1c). Larger tumors were more malignant than smaller tumors (Fig. 1b), especially those larger than 30 mm, but with considerable overlap. Specifically, 66% of benign meningiomas in this sample were smaller than 3 cm (94% < 6 cm), whereas 82% of malignant and borderline malignant ones were larger than 3 cm (22% > 6 cm).

Meningiomas were 2.8 times more frequent in females than in males, but the proportion of borderline malignant and malignant meningiomas was twice as great in men (Fig. 1e). Relative frequency of borderline malignant and malignant meningiomas was slightly higher in patients identified as black or “other” than in patients identified as white (Fig. 1f). The proportion of malignant or borderline malignant tumors was greater for tumors categorized as bilateral than for midline or unilateral tumors (Fig. 1d). This is likely an effect of tumor size, as discussed below. Meningiomas categorized as localizing to “other” regions were more malignant than those localizing exclusively to the cerebral or spinal meninges (Fig. 1g). 84.7% of meningiomas in this “other” group were from ICD-O-3 topography code 71.x (Brain), whereas 13.5% were coded as 72.x (Spinal Cord and Other Central Nervous System), and 1.8% (10 patients) fell under 75.1/75.3 (Pituitary/Pineal glands). In total, 86.3% of 62,844 meningiomas in this sample localized to the cerebral meninges (C70.0), 3.2% to spinal meninges (C70.1), 9.6% to meninges not otherwise specified (C70.9), and only 0.9% to the “other” group. Given the more aggressive behavior of intraparenchymal meningiomas^6^ and the large proportion of meningiomas in this group localizing to C71.x ICD-O-3 topography codes (“Brain”, as opposed to C70.x, “Cerebral Meninges”) we can speculate that this “other” group could, at least in part, consist of intraparenchymal meningiomas.

### Survival

**Fig. 2:**
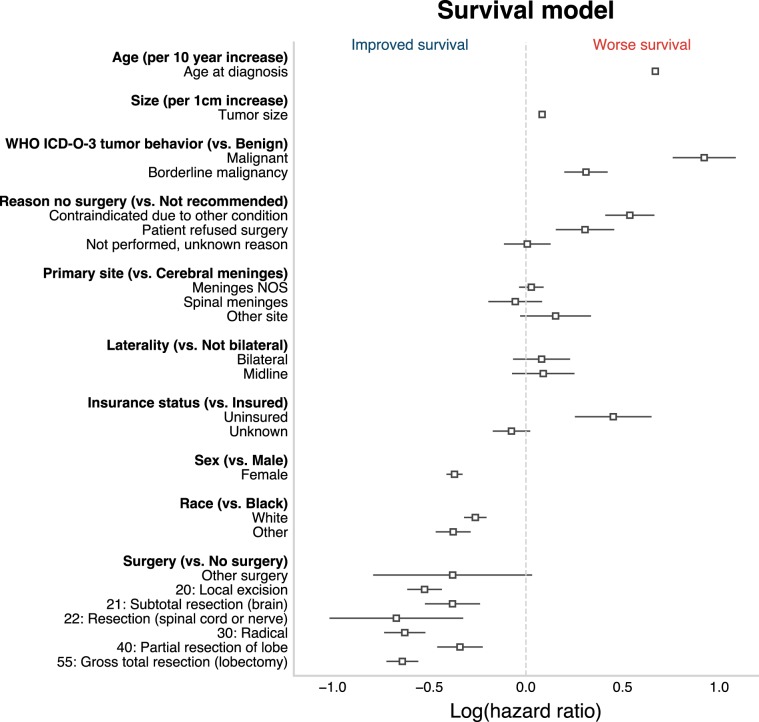
Log hazard ratios for each of the features of the survival model. Negative values indicate proportionally lower probability of death. Positive values indicate proportionally higher probability of death. Error bars represent 95% confidence intervals.

The log hazard ratios of the survival model are presented in Fig. 2. There was an expected effect of age at diagnosis on probability of survival and increased tumor size was associated with worse survival. Malignant tumors predicted worse survival than borderline malignant tumors, and borderline malignant tumors worse survival than benign tumors. At the time of censoring, 76% of patients with a benign meningioma were alive (median age at diagnosis: 66) as compared to 80% of patients with a borderline malignant tumor (median age at diagnosis: 60) and 61% of patients with a malignant tumor (median age at diagnosis: 61). Surgeries coded as “55: Gross total resection”, “30: Radical”, and “22: Resection (spinal cord or nerve)” predicted the greatest improved survival relative to no surgery. Patients who underwent a “40: Partial resection of lobe”, “21: Subtotal resection (brain)”, or other surgery had a relatively smaller improvement in survival. Amongst patients who did not undergo surgery, patients for whom the surgery was contraindicated due to another condition and patients who refused surgery had worse survival relative to patients for whom surgery was not recommended. Patients identified as black had worse survival than non-black patients, males had worse survival than females, and uninsured patients worse survival than insured patients. In the initial analyses, age at diagnosis, tumor size, sex, race, primary tumor site, and laterality were selected as features for both the malignancy and survival models. Additionally, surgical procedure, tumor behavior (if available), insurance status, and reason for no cancer-directed surgery were included in the survival model alone.

### Classifier scoring

**Fig. 3:**
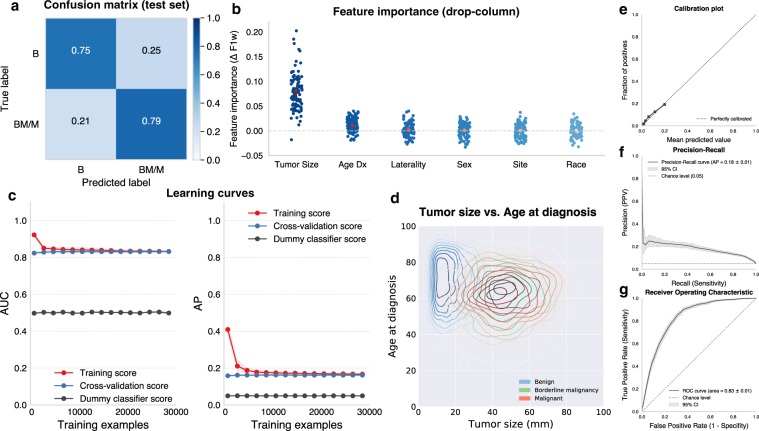
Performance of the malignancy classifier. **a** Confusion matrix illustrating predicted vs. true labels for the malignancy classifier, as evaluated on the test set. Values are normalized across each row. B Benign, BM/M Borderline malignancy/Malignant. **b** Drop-column feature importance showing decrease in classifier performance resulting from dropping a given feature, in decreasing order of importance. The red dot indicates the mean and error bars 95% confidence intervals. **c** Learning curves illustrating training (red line) and cross-validation (blue line) model performance (measured by Area under the receiver operating characteristic curve and Average Precision) as a function of the number of patients used in training the classifier. The point of convergence between the training and cross-validation curves indicates when adding more cases to the training no longer results in an improvement in performance. Shaded outlines represent 1 standard deviation. The gray line represents the performance of a dummy classifier, which randomly generates predictions on the basis of the class distribution in the training set. **d** Bivariate kernel density plot (can be understood as a “2-dimensional histogram”) of tumor size vs. age at diagnosis. **e** Calibration plot, as evaluated on the test set. **f** Precision-recall curve and receiver operating characteristic curve (**g**) for Benign vs. Borderline Malignant/Malignant meningioma classification. For **f**, **g** the gray dashed line indicates chance-level performance and the shaded outline represents the 95% confidence intervals.

Illustration of the performance of the malignancy classifier is presented in Fig. 3. The model was scored on the test dataset consisting of 18,854 randomly assigned patients initially set aside, and a weighted F1 score of 0.82 was obtained. Figure 3a is a confusion matrix showing predicted vs. true class labels, normalized by row, at the selected thresholds used in the app.

The calibration plot (Fig. 3e), precision-recall curve (Fig. 3f), and receiver operating characteristic (ROC) curve (Fig. 3g) are also provided. The calibration diagram plots predicted probabilities against the true observed distribution of each class in the test dataset. The precision-recall curve illustrates precision (positive predictive value) as a function of recall (sensitivity) and is complemented by the receiver operating characteristic (ROC) curve, which illustrates sensitivity and specificity. The average precision was 0.18 (SD: 0.01, chance level: 0.05) and the area under the curve (AUC) was 0.83 (SD: 0.01, chance level: 0.5). At the selected thresholds, we obtained a sensitivity of 0.79 with specificity of 0.75 and a positive predictive value (PPV) of 0.14 with a negative predictive value (NPV) of 0.99. Feature importance is illustrated in Fig. 3b. Tumor size and age at diagnosis were the two most important features in the malignancy model and were the only features retained in the final model. Figure 3d shows the distribution of tumor behavior categories relative to tumor size and age at diagnosis.

Finally, Fig. 3c shows the learning curves (scored for AP and AUC), which illustrate the gain in classification performance attained by increasing the training sample. As a performance baseline, we also plot the classification performance of a “dummy” classifier that randomly generates predictions on the basis of the class distribution in the training set. For both AUC and AP, improvement in model performance plateaus around ~5000–10,000 training examples (i.e., individual patients), after which additional training examples did not improve performance.

**Fig. 4:**
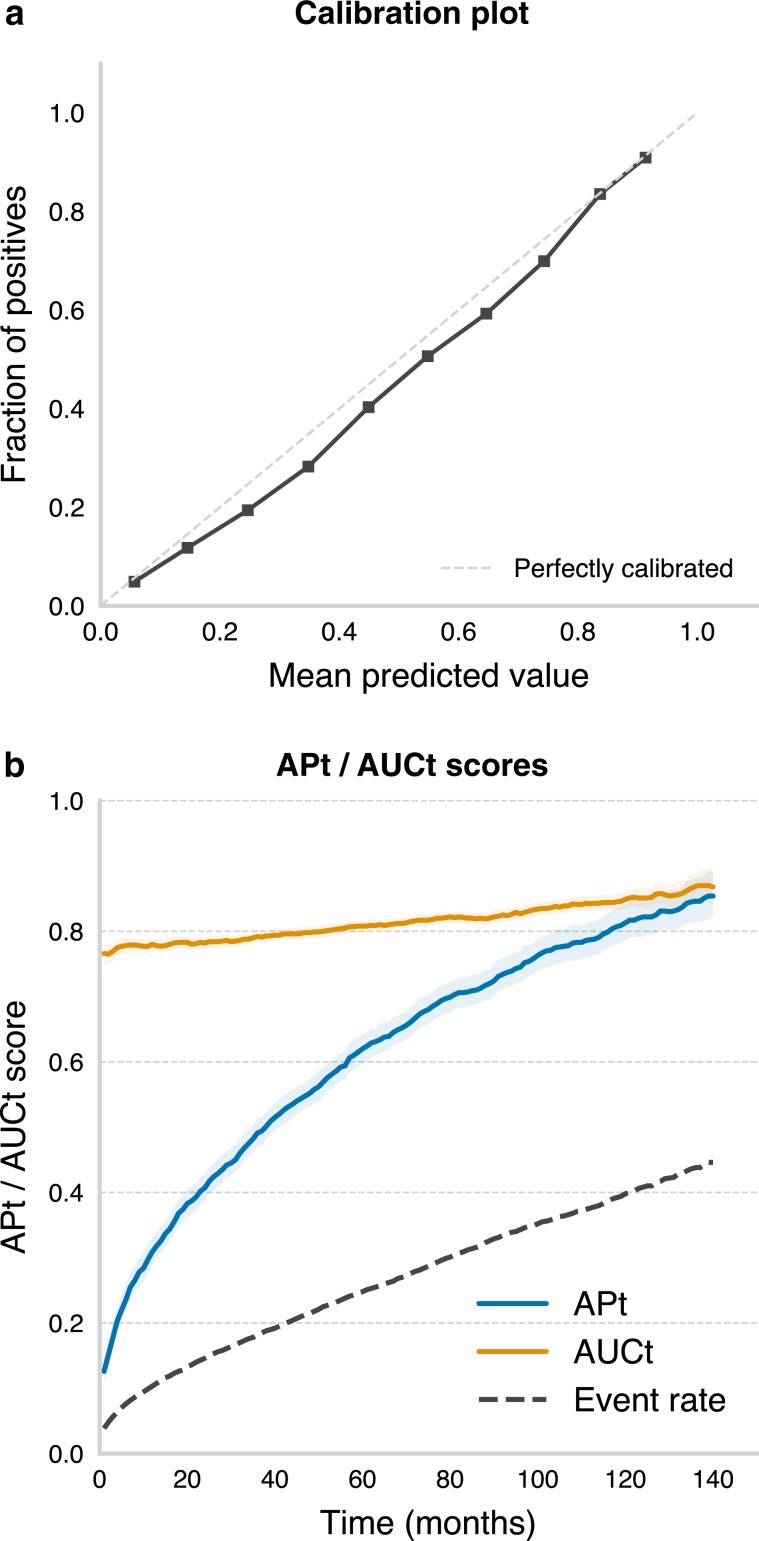
Calibration and performance of the survival model. **a** Survival model calibration plot, as evaluated on the test set. **b** Time-dependent area under the curve (AUCt, yellow line) and average precision (APt, blue line) for the survival model, as evaluated on the test set. The event rate/chance level is represented by the dashed gray line. Shaded outlines represent 95% confidence intervals.

Calibration and performance scoring results for the survival model are shown in Fig. 4. A calibration plot for the survival model, as evaluated on the test set, is shown in Fig. 4a. Figure 4b shows time-dependent average precision (APt) and area under the receiver operating characteristic curve (AUCt)^7,8^ values for the survival model. For 5-year survival, overall APt was 0.62 (95% CI: 0.60-0.64, event rate: 0.25) and AUCt was 0.81 (95% CI: 0.80-0.82). We also obtained a Uno’s C-statistic^9^ of 0.79. Uno’s C in an improvement of Harrel’s concordance index,^10^ which has the added benefit of being independent of the study-specific censoring distribution.^9^ A concordance index of 0.5 represents chance-level performance, whereas a concordance index of 1 indicates perfect performance. In order to assess the generalizability of our classifiers we also subdivided the test set by SEER registry and computed the above reported scores for each registry independently (Table 1).

**Table 1:** Summary of performance metrics for the malignancy and survival models per SEER registry (for all registries with at least 100 cases) as evaluated on the test.

	Malignancy model				Survival model			
SEER registry	F1(w)	AP	Chance level	AUC	Uno’s C	AP (5 y)	Event rate	AUC (5 y)
California excluding SF/SJM/LA	0.80	0.18	0.06	0.82	0.81	0.66	0.26	0.81
Seattle (Puget Sound)	0.88	0.19	0.03	0.88	0.76	0.56	0.22	0.80
Los Angeles	0.78	0.20	0.05	0.83	0.78	0.54	0.22	0.81
New Jersey	0.81	0.18	0.05	0.83	0.79	0.71	0.27	0.83
Kentucky	0.86	0.18	0.04	0.87	0.76	0.54	0.26	0.76
Greater Georgia	0.82	0.15	0.05	0.84	0.72	0.64	0.26	0.80
Detroit (Metropolitan)	0.82	0.15	0.05	0.79	0.76	0.69	0.31	0.81
San Francisco	0.81	0.22	0.06	0.82	0.8	0.60	0.23	0.84
Louisiana	0.82	0.10	0.04	0.80	0.85	0.71	0.30	0.78
Iowa	0.86	0.42	0.07	0.87	0.76	0.64	0.23	0.80
Utah	0.86	0.09	0.02	0.79	0.84	0.67	0.2	0.81
Connecticut	0.80	0.19	0.05	0.84	0.81	0.59	0.21	0.83
Atlanta (Metropolitan)	0.82	0.22	0.05	0.87	0.82	0.58	0.20	0.85
San Jose	0.81	0.21	0.06	0.83	0.77	0.45	0.20	0.79
New Mexico	0.77	0.14	0.05	0.74	0.79	0.61	0.23	0.86
Hawaii	0.78	0.21	0.04	0.88	0.77	0.59	0.27	0.81
Mean	0.82	0.19	0.05	0.83	0.79	0.61	0.24	0.81
SD	0.03	0.07	0.01	0.04	0.04	0.07	0.03	0.03

*F1(w)* weighted F1 score, *AP* average precision, Chance level/event rate baseline performance level for AP, *AUC* area under the receiver operating characteristic curve, *Uno’s C* Uno’s concordance index, *AP(5* *y)* 5-years time-dependent average AP, *AUC(5* *y)* 5-years time-dependent AUC, *SD* standard deviation

## Discussion

We present the development and validation of two classifiers for the prediction of meningioma malignancy and survival. Using only a very limited set of clinical variables, we demonstrate that our models are capable of predicting meaningful clinical outcomes. Previous studies using the SEER database have used various machine learning methods for diagnosis and prognosis purposes in breast^11–15^ and lung cancers,^16,17^ but have not applied these techniques to the SEER data on meningiomas. As compared to classical statistical approaches, the value of predictive modeling is the ability to obtain predictions for individual patients rather than group means. In the framework of levels of evidence for predictive biomarkers proposed by Woo et al.,^18^ the models introduced here fall under the “development” stage and we emphasize the need for future prospective studies and model refinement using imaging and molecular data. The present models represent a valuable performance baseline and proof of concept for future studies to surpass and could, for instance, be used in a Bayesian framework as priors to improve the performance of models developed solely on the basis of imaging or molecular features. We nonetheless believe that meningioma.app provides a unique entry-point for furthering the translatability and transparency of machine learning models, which too often remain impossible for the average clinician to evaluate because of the time and programming knowledge this would require. Our intention here is to allow for clinicians to easily test out the models to provide feedback for improvement and generate interest in the possibilities of such tools. We also hope that this will inspire others to replicate our approach and have therefore made the source code of meningioma.app available under a free open-source license.

We report on the meningioma data up to the November 2017 SEER release, but our observational results are broadly consistent with what has been reported in previous epidemiological literature on meningiomas. From 2004 to 2010, Kshettry et al.^19^ reported that WHO grade II and III meningiomas accounted for 4.2% and 1.2% of newly diagnosed meningiomas, respectively. Likewise, after exclusions, we identified a total of 62,844 meningiomas for the period between 2004 and 2015, of which 4.0% were coded as borderline malignant and 0.9% as malignant. As in our previous work, we found that while pediatric meningiomas may include relatively more aggressive subtypes, young age is overall associated with reduced all-cause mortality.^4^ In regard to sex, across all ages overall, meningiomas were more frequent in females than in males, but malignancy^19^ and mortality^3,20,21^ were greater in males than females. Also concordant with previous reports, black race and larger tumor size were found to be adverse prognostic factors.^3,22,23^ Regarding lateralization, we found that the proportion of malignant/borderline malignant tumors was greater for bilateral meningiomas. This effect is presumably explained by size as bilateral tumors were on average larger (41.5 mm) than midline (30.0 mm) or unilateral (27.2 mm) tumors. Gross total resection was also found to be a strong predictor of longer survival.^3,21,23^ Additionally, in patients who did not undergo surgery, we found differences in survival based on the reason why no cancer-directed surgery was performed, with relatively worse survival in patients who refused surgery or for whom surgery was contraindicated due to another condition as compared to patients for whom surgery was not recommended. We also found, as expected, that uninsured patients had worse survival than insured patients.

As compared to previous studies of meningiomas in SEER, our present study is chiefly differentiated by the application of statistical learning methods. Specifically, we trained an ensemble voting classifier using a random undersampling procedure inspired by the Balanced Random Forest algorithm^24^ and proportional hazards models^25,26^ to predict malignancy and survival. Ensemble classifiers often outperform any classifier used independently and also can help reduce the risk of overfitting the training data.^27^ In this study, we found that the method indeed did help produce a better calibrated model while also marginally improving classification performance over either balanced random forest or balanced logistic regression used alone (Supplementary Figs. 1 and 2). The results in Fig. 3e indicate that the model is well-calibrated (e.g., a predicted probability of 20% that a meningioma is non-benign suggests a true 20% chance that the meningioma is indeed non-benign), but does not make predictions with high probability values. This is consistent with the classification accuracy of the model, but is also in part due to very imbalanced base class distribution in this dataset; any randomly selected meningioma has about a 95% chance of being benign and, conversely, only a 5% chance of being non-benign.

**Fig. 5:**
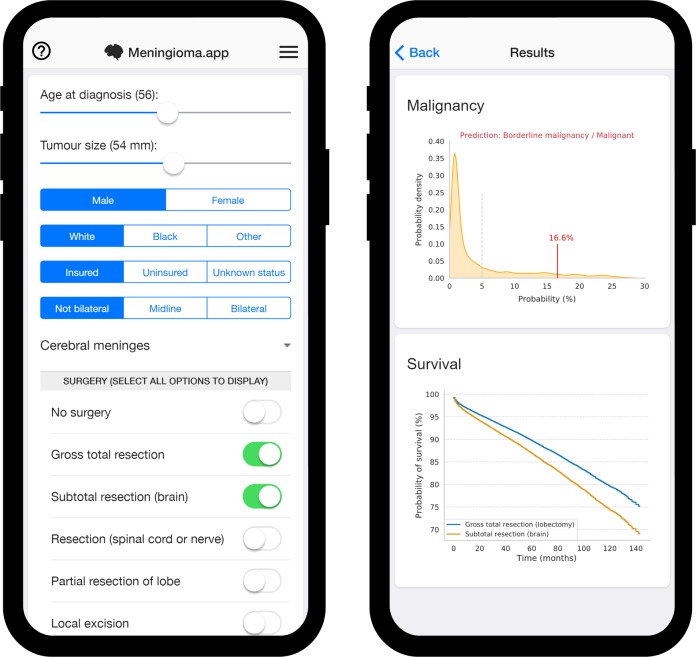
Example predicted malignancy and survival curves for an insured 56-year-old white man with a unilateral 54 mm wide meningioma localizing to the cerebral meninges. Try the app at www.meningioma.app.

An intrinsic advantage of ensemble classifiers is the ability not only to provide a binary prediction of the predicted outcome, but also to provide probability estimates by calculating the proportion of votes in the ensemble (e.g., if 50 of 100 base classifiers in the ensemble predict one outcome, the predicted probability estimate is 50%). This is illustrated together with individualized survival curves for an example 56-year-old man in the screenshots of our app shown in Fig. 5. A second calibration step was however necessary to provide well-calibrated probability estimates for the malignancy classifier, as discussed in the Methods below. One important consideration is that the provided individualized survival curves should only be used to estimate survival of a patient for whom a specific treatment course has been decided and not to guide treatment decisions for that specific patient. While perhaps a seemingly subtle interpretation difference, the second use would likely be invalid due to probable patient selection effects in SEER (e.g., the patients who did not undergo surgery are not the same patients who received a gross total resection). Our smartphone optimized web app (www.meningioma.app), allows inputting basic clinical details for any new patient to obtain straightforward predictions of malignancy and survival. The details entered into the app are run through exactly the same models described in this paper, but without the need for any advanced technical or programming knowledge.

Previous studies have used classifiers for the detection and grading of meningiomas and other CNS tumors, but these have almost exclusively focused on MRI^28,29^ or histopathological^30^ imaging characteristics to drive their predictions. We also extend classical survival analysis methods to the machine learning framework and demonstrate how proportional hazard ratios can be used to create individualized patient-specific survival curves (also illustrated in Fig. 5). While predictive modeling of imaging and molecular-genetic profiles should undoubtedly form part of the effort for more accurate diagnostic and prognostic tools,^31^ we demonstrate here that the informational value to be gained from even the simplest of clinical variables is not to be ignored. Moreover, we position the value of such a tool as being of particular relevance at the pre-biopsy/surgery stage, which is of particular interest in the case of meningiomas where only roughly half of tumors are microscopically confirmed.^22^ It is also worth noting that the sample size of these previous studies was on an entirely different scale, ranging from dozens to a few hundred patients at most. Rather than competing with these prior models, what we hope to highlight here is the potential value of combining models trained on large epidemiological datasets with classifiers trained on smaller but richer datasets. Further improvements to model performance will be needed before clinical translatability can be achieved. While marginal improvements might still be achievable with the current dataset by refining the models themselves, a larger challenge for translatability lies in collecting and curating large multimodal datasets for training and validation against clinical outcomes.

With ever decreasing storage costs and the advent of open databasing solutions for genetic^32^ and neuroimaging data,^33,34^ the possibility of expanding the scope of national cancer registries for large-scale inclusion of de-identified source data will lead the way for the next generation of predictive models. Recent large-scale projects for population genotyping and brain imaging such as the UK biobank represent a significant opportunity in this regard.^35,36^ Additionally, efforts to provide curation, as well as streamlined consent^37^ and de-identification^38,39^ of data from electronic medical records and picture archiving communication systems, are another important step in this direction.^40–43^ Allowing for the wealth of patient data already being recorded for routine care to be used to advance predictive disease modeling has the potential to simplify specific aspects of clinical decision making, as well as improve diagnostic and prognostic accuracy for future patients. We also highlight the need to expand outcome variable reporting beyond survival. Indeed, functional outcomes and quality of life are also key to informed clinical decision making and patient counseling. In the case of meningiomas, surgery for benign tumors is frequently undertaken to treat comorbid seizures or other neurological symptoms. We could therefore imagine predictive models being developed to determine which patients are more likely to benefit from such interventions. Likewise, we could foresee training models to learn patterns of association between certain tumor features (e.g., size and location) and treatment variables (surgery type, adjunctive therapy), and the probability of specific neurological complications.

There are inherent limitations to any study of retrospective registry-based data, such as selection and reporting biases. We have described these in detail in previous work.^44–47^ Nonetheless, one of the benefits of the present study in this regard was the random assignment of 30% of patients to a “test” dataset, which was sequestered until the final models were developed. This allowed for pseudo-prospective evaluation of our models and therefore reduced bias in the scoring of model performance. While we demonstrated good generalizability of the model across SEER registries, the true test of these models will have to come from replication with prospective, multi-registry, and international patient cohorts. Also, given the poor discrimination between borderline malignant and malignant meningiomas (Fig. 3d) based on the limited set of clinical variables available in SEER, we opted to focus current analyses on binary classification between benign and non-benign tumors. We consider the ability to differentiate these two categories to be the more important question from a clinical perspective since, as previously reported by Dolecek et al.,^22^ only 29% of borderline malignant and 31% of malignant meningiomas received no initial treatment, as compared to 60% of benign meningiomas. Future work with richer datasets should, however, attempt to distinguish between these categories.

Regarding treatment variables, only surgery was investigated and we did not include radiotherapy or chemotherapy as features of interest in the survival classifier because there are substantive concerns with these data in SEER.^48^ Starting with the November 2016 data submission, these data have been removed from the main SEER research databases. We do, however, emphasize the need for radiotherapy in particular to be investigated with another dataset. Also, Simpson grading is not available in SEER and some heterogeneity is therefore expected in the gross total resection group.^49^ We also excluded the small percentage of patients who had a second meningioma recorded in SEER so as not to bias scoring of the model (i.e., each training or testing example was one patient). It would, however, be valuable to predict outcomes in these patients with subsequent meningiomas—or any second cancer, whether occurring prior to or after the meningioma. Finally, this study remains, at least in part, a proof of concept of what can be achieved with predictive modeling of cancer registry data. We fully realize that more powerful models will need to integrate radiographic and molecular features in their predictions, and hope to update meningioma.app with such models in future work.

We report the development and validation of predictive models of meningioma malignancy and associated survival. On the basis of a very limited set of clinical variables such as age, sex, and tumor size, our models are shown to be capable of predicting individual-patient outcomes. Our modeling approach provides complementary information to previous epidemiological reports and could lead to the development of new practical diagnostic and prognostic tools in oncology. Beyond the traditional paper-and-pencil nomograms, we provide an original open-source smartphone and web application to illustrate the translation of complex nonlinear predictive models to real-world practice. In particular, the ability of our statistical learning models and app to provide individual-specific predicted survival curves could be valuable for patient counseling.

## Methods

### Participants

**Fig. 6:**
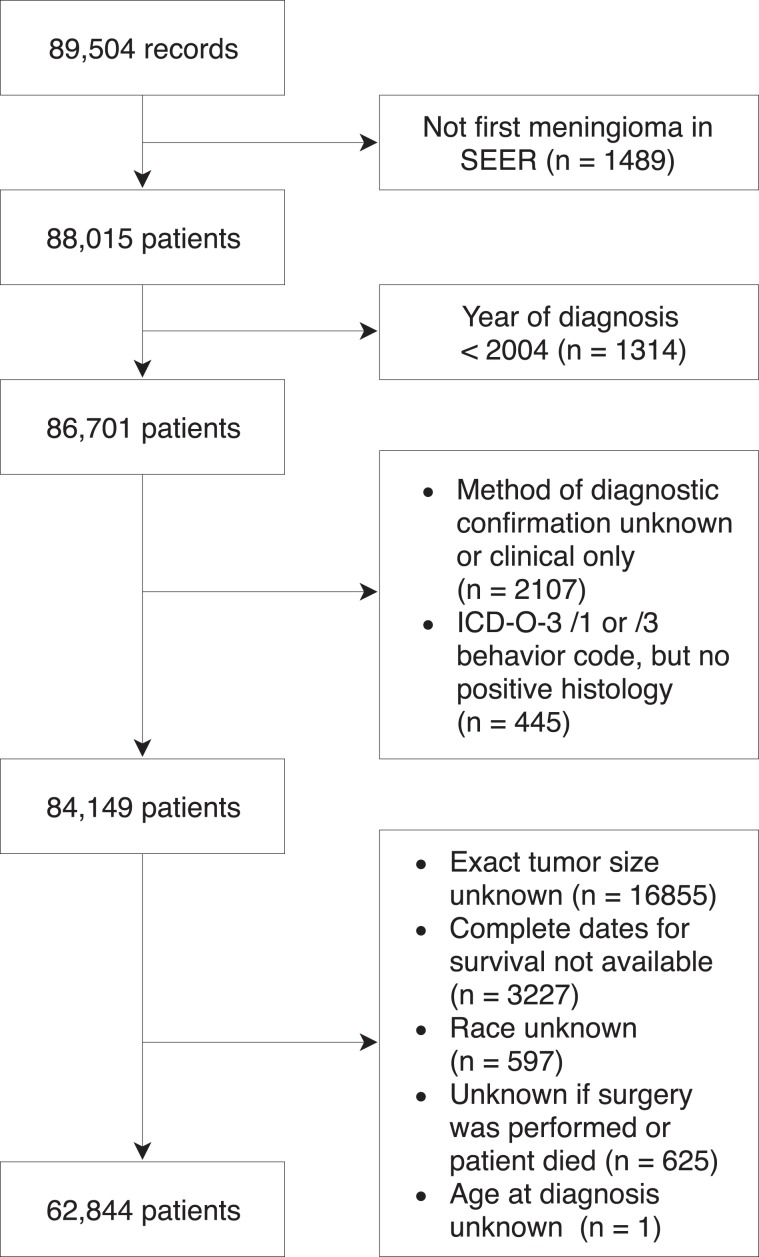
Flow diagram illustrating criteria for patient inclusion.

The latest SEER data release (November 2017) was queried using SEER*Stat v8.3.5 for all cases of meningioma (WHO ICD-O-3 histology codes 9530-9539) recorded in the brain and spinal cord. A complete description of the SEER*Stat search query is provided in the supplementary information (Supplementary Note 1). The data included patients diagnosed between 2004 and 2015 across 18 registries in 13 states. Only the first meningioma recorded in SEER for each patient was included in analyses. In total, 88,015 patients were initially identified. Patients diagnosed prior to 2004 were excluded because their diagnosis predates the passage of Public Law 107–260, which mandated the collection of non-malignant tumors.^22^ Patients for whom the method of diagnostic confirmation was unknown or clinical only were also excluded. Moreover, all ICD-O-3/1 (borderline malignant) and/3 (malignant) meningiomas without positive histological diagnosis were excluded. We also excluded meningiomas recorded as being larger than 150 mm as such cases are extremely rare and more likely represent coding errors in SEER (e.g., an “803 mm” meningioma). In addition to these exclusion criteria, we excluded case listings for which features (age, tumor size, race, tumor site, surgery) or outcome variables (malignancy, survival) of interest were not available. Exclusion criteria are illustrated in Fig. 6. After exclusions, the final number of patients included in analyses was 62,844. As SEER contains no personally identifiable information and this study relied exclusively on secondary use of observational epidemiological data from a public national database, our institutional research ethics board deemed this study to be exempt from review. Transparent Reporting of a multivariable prediction model for Individual Prognosis Or Diagnosis (TRIPOD) reporting guidelines were implemented in this manuscript.^50^

### Feature selection

Descriptive statistics were computed and exploratory data analysis was performed to identify potential features (i.e., predictor variables) for inclusion in the machine learning models. Selection criteria for features included data availability (variables with large numbers of missing points were excluded) and discriminatory capability in relation to the two outcomes of interest—malignancy and survival. Malignancy was defined as per WHO ICD-O-3 histology and behavior codes. ICD-O-3 behavior codes were used as WHO grade is not consistently available for meningiomas in SEER.^19,23^ Previous studies have used the following correspondence from WHO grade to ICD-O-3 histology and behavior codes: WHO I: 9530/0, 9531/0, 9532/0, 9533/0, 9534/0, 9537/0; WHO II: 9530/1, 9531/1, 9532/1, 9533/1, 9534/1, 9537/1, 9538/1, 9539/1; WHO III: 9530/3, 9531/3, 9532/3, 9533/3, 9534/3, 9535/3, 9537/3, 9538/3, 9539/3.^19,23^ We have opted here to display the original ICD-O-3 labels as they are reported in SEER (i.e., /0: benign, /1: borderline malignancy, /3: malignant).

All-cause mortality was used in survival analyses because cause-specific mortality is not reliably available across all meningioma cases in SEER.^4^ Moreover, as demonstrated in Fig. 2, treatment and clinical variables other than age clearly impact all-cause survival. We also obtained a Uno’s C of 0.70 for a model excluding malignancy and age at diagnosis, as compared to a Uno’s C of 0.79 for the model including all features. Supplementary Fig. 3 additionally illustrates AUCt and APt when age and malignancy are excluded from the survival model. Survival was defined on the basis of the “Survival months” variable in SEER, which is calculated on the basis of the date at diagnosis and date at last contact.^5^ Censoring was based on the “Vital status recode” variable in SEER. Features with low-frequency classes were recoded into more general classes where appropriate in order to have sufficient examples for training and cross-validation. Features were also recoded when it was found that two or more classes did not provide additional information in respect to the outcome variable (e.g., patients with left vs right sided meningiomas had equivalent survival). Primary tumor site was recoded by ICD-O-3 topography codes as either “cerebral meninges” (C70.0), “spinal meninges” (C70.1), “meninges not otherwise specified” (C70.9), and “other”. Race, as defined in the SEER database, was recoded into “white”, “black”, and “other” groups. Tumor laterality was recoded as “bilateral” (e.g., large meningiomas extending over both hemispheres), “midline” (e.g., falcine meningiomas), or “not bilateral”. The original SEER coding for surgical procedures was preserved except for “10: Tumor destruction NOS”, “50: Surgery stated to be “debulking”, and “90: Surgery, NOS” codes, which were recoded as “other surgery” because they accounted in total for only 0.7% of all surgically treated cases.

### Classifier design and validation

Of the 62,844 included patients, 30% (18,854) were randomly selected and set aside for use as a test (validation) dataset, whereas the data of the remaining 43,990 patients were used for training (development) and cross-validation. In all, 13,197 of these 43,990 cases were used for cross-validation and initial model exploration (Supplementary Fig. 1). The use of a true “test” set allows for unbiased estimation of model performance, which cannot be achieved through cross-validation alone since human or automated optimization of models and model parameters inherently biases scoring towards artificially inflated classification accuracy.^51^ Two separate classifiers were trained and tested, one for each of the outcome variables of interest. Preprocessing, hyperparameter optimization, cross-validation, and scoring were performed using the scikit-learn Python module.^52^

For the malignancy model, a voting ensemble classifier (BLR-RF) combining balanced logistic regression and balanced random forest base models was implemented. This BLR-RF classifier implements a random undersampling procedure akin to that of the Balanced Random Forest (BRF)^24^ algorithm whereby each base classifier in the ensemble is trained on a randomly selected class-balanced subsample of the training dataset. The imbalanced-learn Python package^53^ was used to perform the random undersampling and the MLxtend package to build the ensemble voting classifier.^54^ As compared to BRF, we found that, in this dataset, our BLR-RF classifier provided better probability calibration and was less prone to overfitting (Supplementary Fig. 1). The BRF algorithm is an extension of the popular random forest algorithm,^55^ which builds ensembles of decision trees and uses a voting procedure to output the overall classification decision. Both our BLR-RF classifier and BRF are distinguished from standard random forests by additionally resampling data within random bootstrap samples (smaller samples randomly drawn from the training data) to address the problem of class imbalance.^24^ We found this procedure to be effective in this highly imbalanced dataset (i.e., ~95% of meningiomas were benign), as compared to regular logistic regression or random forests (Supplementary Fig. 1). In the implementation used in the present study, we resampled the non-minority classes (i.e., all non-malignant tumors) so that each bootstrap sample contained roughly equal numbers of benign and non-benign tumors. In order to improve model calibration, we applied a second probability calibration step using Platt scaling^56^ with a subset of data not used in the initial training (Supplementary Fig. 1).

Hyperparameter optimization was performed using 1000 iterations of randomized^57^ fivefold stratified K-fold cross-validation using the weighted F1 score, as implemented in scikit-learn,^52^ as the primary scoring metric. We selected this weighted F1 score as the scoring metric for training the model as it penalizes misclassifications of the minority class (i.e., non-benign meningiomas) to a greater extent, which is critical in this very imbalanced dataset (e.g., we could obtain 95% accuracy simply by classifying all meningiomas as benign). The F1 score ranges from 0 (worst) to 1 (best) and is defined as the harmonic mean of precision (PPV) and recall (sensitivity). It is better suited than accuracy or area under the receiver operating characteristic curve for measuring classifier performance in imbalanced datasets.^58^ Candidate models were also evaluated using confusion matrices obtained from the cross-validation set. Confusion matrices are a simple way to represent true vs. predicted classes and calculate rates of true and false positives and negatives. For the survival model, we used the implementation of Cox’s proportional hazards model in the lifelines Python package.^26^ This model is suited for working with censored survival data and has the benefit of providing easily interpretable prediction probabilities.

Model scoring for the malignancy model was performed using a series of metrics, including the F1 score and confusion matrices, as described above, but also precision-recall and receiver operating characteristic (ROC) curves, which can also be summarized by the average precision (AP) and AUC metrics. AUC tends to provide overly optimistic estimates of performance in imbalanced datasets and it is therefore also useful to evaluate the precision-recall curve in these cases.^59^ Average precision is prevalence-dependent and should therefore be evaluated in the context of the baseline population prevalence. We report chance-level values to illustrate this baseline. For the survival model, we report time-dependent average precision (APt)^7^ and area under the curve (AUCt)^60^ values, using the R implementation in the APtools package,^8^ as well as Uno’s C-statistic.^9^ We used the implementation of Uno’s C provided in the scikit-survival Python package.^61^ In addition to Uno’s C, time-dependent AP and AUC values provide a useful complement for model evaluation. APt has been suggested to be of particular value for assessing low-probability events,^8^ but is also dependent on prevalence and should therefore be evaluated against the baseline event rate at each time point. In addition to the above metrics, we also provide calibration plots of predicted vs. observed risk. Confidence intervals were computed using bootstrap resampling of the test set with 1000 iterations for precision-recall, ROC, APt, and AUCt values.

### Reporting summary

Further information on experimental design is available in the Nature Research Reporting Summary linked to this paper.

## Supplementary information

Supplementary Information

Nature Research Reporting Summary

## Data Availability

All data used in this study are available for download through the SEER program.

## Code availability

The source code for meningioma.app is available for download at https://github.com/jeremymoreau/meningioma.

## References

[1] Ostrom QT (2017). CBTRUS statistical report: primary brain and other central nervous system tumors diagnosed in the United States in 2010-2014. Neuro. Oncol..

[2] Porter KR, McCarthy BJ, Freels S, Kim Y, Davis FG (2010). Prevalence estimates for primary brain tumors in the United States by age, gender, behavior, and histology. Neuro. Oncol..

[3] Aizer AA (2015). Extent of resection and overall survival for patients with atypical and malignant meningioma. Cancer.

[4] Dudley RWR (2018). Pediatric versus adult meningioma: comparison of epidemiology, treatments, and outcomes using the Surveillance, Epidemiology, and End Results database. J. Neurooncol..

[5] National Cancer Institute. Overview of the SEER Program. Available at: https://seer.cancer.gov/about/overview.html. Accessed in 2019.

[6] Ohba S, Abe M, Hasegawa M, Hirose Y (2016). Intraparenchymal meningioma: clinical, radiologic, and histologic review. World Neurosurg..

[7] Yuan Y, Su W, Zhu M (2015). Threshold-free measures for assessing the performance of medical screening tests. Front. Public Health.

[8] Cai, H., Yuan, Y., Zhou, Q. M. & Li, B. APtools: Average Positive Predictive Values (AP) for Binary Outcomes and Censored Event Times. R package version 6.8.8. https://CRAN.R-project.org/package=APtools (2018).

[9] Uno H, Cai T, Pencina MJ, D’Agostino RB, Wei LJ (2011). On the C-statistics for evaluating overall adequacy of risk prediction procedures with censored survival data. Stat. Med..

[10] Harrell FE, Lee KL, Mark DB (1996). Multivariable prognostic models: issues in developing models, evaluating assumptions and adequacy, and measuring and reducing errors. Stat. Med..

[11] Shukla N, Hagenbuchner M, Win KT, Yang J (2018). Breast cancer data analysis for survivability studies and prediction. Comput. Methods Prog. Biomed..

[12] Kate RJ, Nadig R (2017). Stage-specific predictive models for breast cancer survivability. Int. J. Med. Inform..

[13] Lotfnezhad Afshar H, Ahmadi M, Roudbari M, Sadoughi F (2015). Prediction of breast cancer survival through knowledge discovery in databases. Glob. J. Health Sci..

[14] Wang, X., Hershman, D. L. & Neugut, A. I. Using machine learning, general regression, and Cox proportional hazards regression to predict the effectiveness of treatment in patients with breast cancer. *AMIA Annu. Symp. Proc*. 1133. https://www.ncbi.nlm.nih.gov/pubmed/17238752 (2006). PMC1839432 17238752

[15] Shin H, Nam Y (2014). A coupling approach of a predictor and a descriptor for breast cancer prognosis. BMC Med. Genomics.

[16] Lynch CM, van Berkel VH, Frieboes HB (2017). Application of unsupervised analysis techniques to lung cancer patient data. PLoS ONE.

[17] Lynch CM (2017). Prediction of lung cancer patient survival via supervised machine learning classification techniques. Int. J. Med. Inform..

[18] Woo C-W, Chang LJ, Lindquist MA, Wager TD (2017). Building better biomarkers: brain models in translational neuroimaging. Nat. Neurosci..

[19] Kshettry VR (2015). Descriptive epidemiology of World Health Organization grades II and III intracranial meningiomas in the United States. Neuro. Oncol..

[20] Westwick HJ, Shamji MF (2015). Effects of sex on the incidence and prognosis of spinal meningiomas: a Surveillance, Epidemiology, and End Results study. J. Neurosurg. Spine.

[21] Amsbaugh M (2016). Patterns of care and outcomes of adjuvant radiotherapy for meningiomas: a surveillance, epidemiology, and end results and medicare linked analysis. Cureus.

[22] Dolecek TA (2015). Epidemiology of meningiomas post-Public Law 107-206: The Benign Brain Tumor Cancer Registries Amendment Act. Cancer.

[23] Garzon-Muvdi T., Yang W., Lim M., Brem H., Huang J. (2017). Atypical and anaplastic meningioma: outcomes in a population based study. Journal of Neuro-Oncology.

[24] Chen, C, Liaw, A. & Breiman, L. *Using Random Forest to Learn Imbalanced Data*. Vol. 110 1–12 (University of California, Berkeley, 2004).

[25] Cox DR (1972). Regression models and life-tables. J. R. Stat. Soc. Ser. B Stat. Methodol..

[26] Davidson-Pilon, C. et al. *CamDavidsonPilon/lifelines: v0.20.3*. (2019). 10.5281/zenodo.2604107.

[27] Dietterich Thomas G. (2000). Ensemble Methods in Machine Learning. Multiple Classifier Systems.

[28] Yan P-F (2017). The potential value of preoperative MRI texture and shape analysis in grading meningiomas: a preliminary investigation. Transl. Oncol..

[29] Sachdeva, J., Kumar, V., Gupta, I., Khandelwal, N. & Ahuja, C. K. Multiclass brain tumor classification using GA-SVM. in *2011 Developments in E-systems Engineering* 182–187 (Institute of Electrical and Electronics Engineers, 2011).

[30] Qureshi, H., Sertel, O., Rajpoot, N., Wilson, R. & Gurcan, M. Adaptive Discriminant Wavelet Packet Transform and Local Binary Patterns for Meningioma Subtype Classification. in *Medical Image Computing and Computer-Assisted Intervention—MICCAI 2008* 196–204 (Springer, Berlin, Heidelberg, 2008). 10.1007/978-3-540-85990-1_24 18982606

[31] Nowosielski M (2017). Diagnostic challenges in meningioma. Neuro. Oncol..

[32] Cerami E (2012). The cBio cancer genomics portal: an open platform for exploring multidimensional cancer genomics data. Cancer Disco..

[33] Clark K (2013). The Cancer Imaging Archive (TCIA): maintaining and operating a public information repository. J. Digit. Imaging.

[34] Niso G (2016). OMEGA: the open MEG archive. Neuroimage.

[35] Bycroft C (2018). The UK Biobank resource with deep phenotyping and genomic data. Nature.

[36] Miller KL (2016). Multimodal population brain imaging in the UK Biobank prospective epidemiological study. Nat. Neurosci..

[37] Gagnon J, Leggett JA, Richard C, Lussier M-T (2014). Facilitating informed consent for EMR research in Quebec. Can. Fam. Physician.

[38] Das S (2016). The MNI data-sharing and processing ecosystem. Neuroimage.

[39] Das S (2016). Cyberinfrastructure for open science at the Montreal Neurological Institute. Front. Neuroinform.

[40] Keshavjee K (2014). Getting to usable EMR data. Can. Fam. Physician.

[41] Owens Brian (2018). Family doctors call for guaranteed access to EMR data for research and quality improvement. Canadian Medical Association Journal.

[42] Jensen PB, Jensen LJ, Brunak S (2012). Mining electronic health records: towards better research applications and clinical care. Nat. Rev. Genet..

[43] Rajkomar A (2018). Scalable and accurate deep learning with electronic health records. NPJ Digital Med..

[44] Dudley RWR (2015). Pediatric low-grade ganglioglioma: epidemiology, treatments, and outcome analysis on 348 children from the surveillance, epidemiology, and end results database. Neurosurgery.

[45] Dudley RWR (2015). Pediatric choroid plexus tumors: epidemiology, treatments, and outcome analysis on 202 children from the SEER database. J. Neurooncol..

[46] Hankinson TC (2012). Limited utility despite accuracy of the national SEER dataset for the study of craniopharyngioma. J. Neurooncol..

[47] Hankinson TC (2016). Short-term mortality following surgical procedures for the diagnosis of pediatric brain tumors: outcome analysis in 5533 children from SEER, 2004-2011. J. Neurosurg. Pediatr..

[48] Noone A-M (2016). Comparison of SEER treatment data with medicare claims. Med. Care.

[49] Stessin AM (2012). Does adjuvant external-beam radiotherapy improve outcomes for nonbenign meningiomas? A Surveillance, Epidemiology, and End Results (SEER)-based analysis. J. Neurosurg..

[50] Collins GS, Reitsma JB, Altman DG, Moons KGM (2015). Transparent reporting of a multivariable prediction model for Individual Prognosis or Diagnosis (TRIPOD): the TRIPOD statement. Ann. Intern. Med..

[51] Ripley, B. D. *Pattern Recognition and Neural Networks*. (Cambridge University Press, 2007).

[52] Pedregosa F (2011). Scikit-learn: machine learning in Python. J. Mach. Learn. Res..

[53] Lemaître G, Nogueira F, Aridas CK (2017). Imbalanced-learn: a Python toolbox to tackle the curse of imbalanced datasets in machine learning. J. Mach. Learn. Res..

[54] Raschka S (2018). MLxtend: providing machine learning and data science utilities and extensions to Python’s scientific computing stack. J. Open Sour. Softw..

[55] Breiman L (2001). Random Forests. Mach. Learn..

[56] Platt, J. C. Probabilistic Outputs for Support Vector Machines and Comparisons to Regularized Likelihood Methods. in *Advances in Large Margin Classifiers* 61–74 (MIT Press, 1999).

[57] Bergstra J, Bengio Y (2012). Random search for hyper-parameter optimization. J. Mach. Learn. Res..

[58] Liu M (2018). Cost-sensitive feature selection by optimizing F-measures. IEEE Trans. Image Process.

[59] Ozenne B, Subtil F, Maucort-Boulch D (2015). The precision–recall curve overcame the optimism of the receiver operating characteristic curve in rare diseases. J. Clin. Epidemiol..

[60] Heagerty PJ, Lumley T, Pepe MS (2000). Time-dependent ROC curves for censored survival data and a diagnostic marker. Biometrics.

[61] Pölsterl, S., Navab, N. & Katouzian, A. in *Machine Learning and Knowledge Discovery in Databases.* 243–259 (Springer International Publishing, 2015).

