# Supplementary material for: Individual-patient prediction of meningioma malignancy and survival using the Surveillance, Epidemiology, and End Results database
Source: NPJ Digit Med. 2020 Jan 30;3:12. doi: 10.1038/s41746-020-0219-5 (PMC6992687; doi:10.1038/s41746-020-0219-5)
Supplement: Supplementary file 1 — Supplementary Information [file 41746_2020_219_MOESM1_ESM.pdf]

Supplementary Information

Supplementary Figures

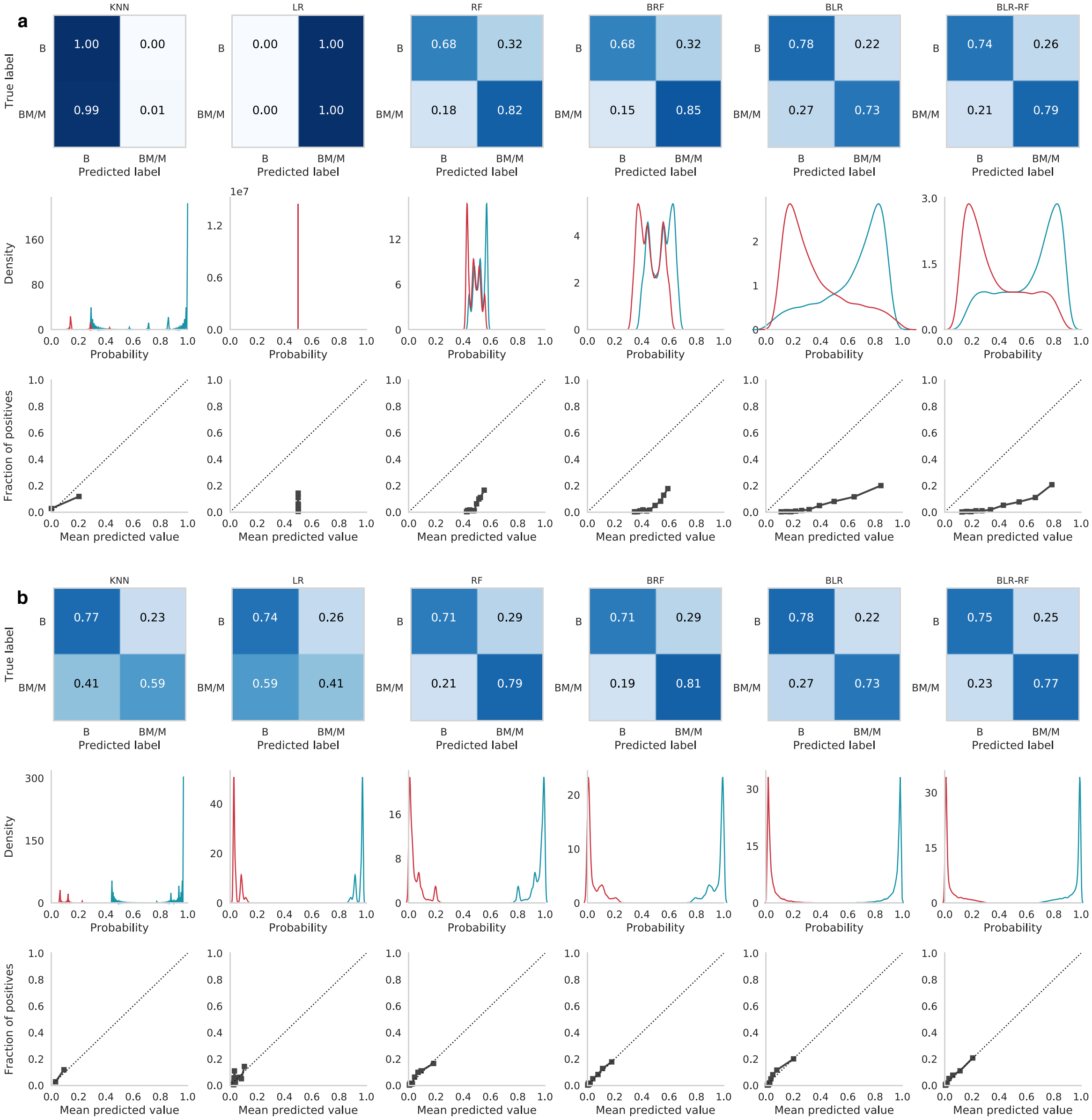

Supplementary Fig. 1. Exploratory model comparison for initial models after hyperparameter optimization but prior to probability calibration **(a)**, and after calibration **(b)**. KNN: K-Nearest Neighbors, LR: Logistic regression, RF: Random Forests, BRF: Balanced Random Forest, BLR: Balanced Logistic Regression, BLR-RF: Balanced Logistic Regression-Random Forests. For (a) and (b), the first row contains confusion matrices illustrating predicted vs. true labels, as evaluated on the test set. Values are normalized across each row. B: Benign, BM/M: Borderline malignancy/Malignant. The second row consists of kernel density plots illustrating the distribution of predicted probabilities for the benign (blue) and non-benign (red) meningioma classes. The third row consists of calibration diagrams plotting predicted probabilities against the true observed distribution of each class in the test dataset.

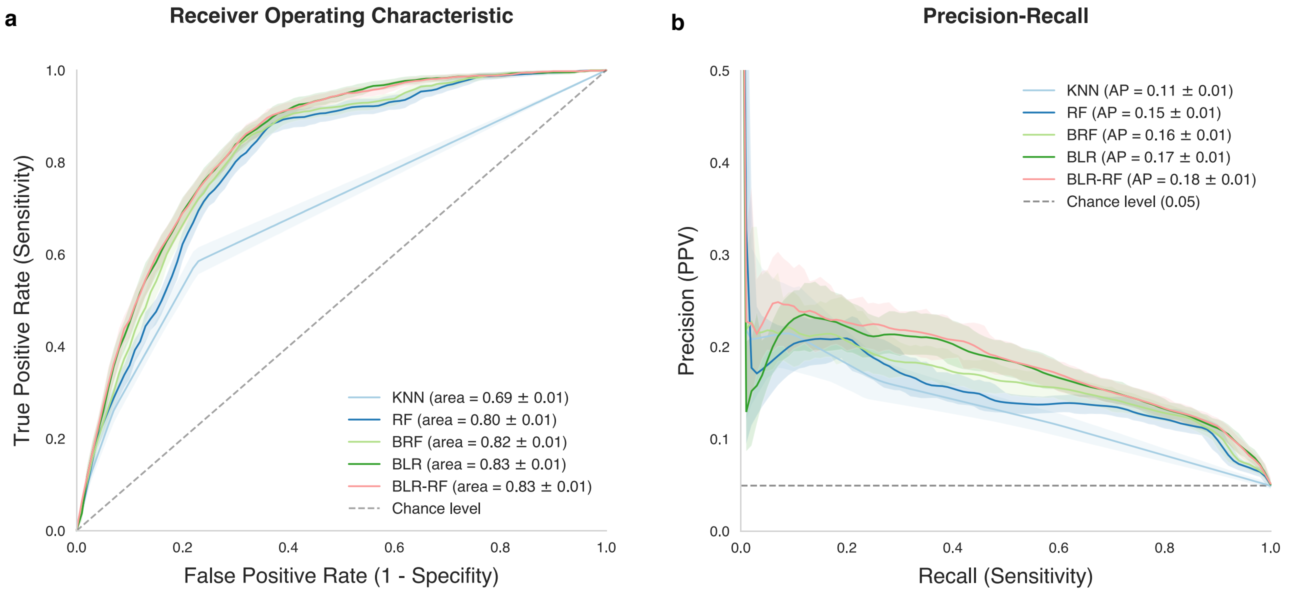

Supplementary Fig. 2. Receiver Operating Characteristic curve **(a)** and Precision-Recall curve **(b)** for Benign vs. Borderline Malignant/Malignant meningioma classification. The grey dashed line indicates chance level performance and the shaded outlines represents 95% confidence intervals. KNN: K-Nearest Neighbors, LR: Logistic regression, RF: Random Forests, BRF: Balanced Random Forest, BLR: Balanced Logistic Regression, BLR-RF: Balanced Logistic Regression-Random Forests

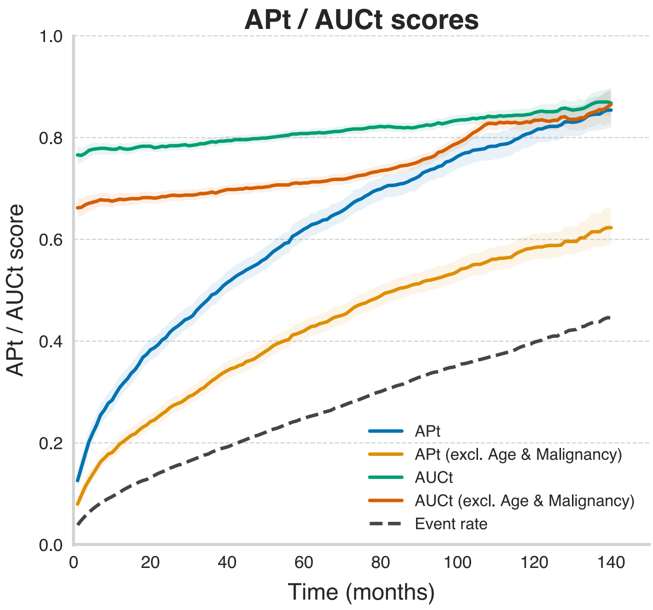

Supplementary Fig. 3. Time-dependent area under the curve (AUCt) and average-precision (APt) for the survival model including or excluding age and malignancy as features of interest, as evaluated on the test set. The event rate/chance level is represented by the dashed grey line. Shaded outlines represent 95% confidence intervals.

Supplementary Notes

Supplementary Note 1. SEER*Stat search query.

Database Name:
Incidence - SEER 18 Regs Research Data + Hurricane Katrina Impacted Louisiana Cases, Nov 2017 Sub (1973-2015 varying)

Selection:
{Site and Morphology.Primary Site - labeled} = 'C70.0-Cerebral meninges','C70.1-Spinal meninges','C70.9-Meninges, NOS','C71.0-Cerebrum','C71.1-Frontal lobe','C71.2-Temporal lobe','C71.3-Parietal lobe','C71.4-Occipital lobe','C71.5-Ventricle, NOS','C71.6-Cerebellum, NOS','C71.7-Brain stem','C71.8-Overlapping lesion of brain','C71.9-Brain, NOS','C72.0-Spinal cord','C72.1-Cauda equina','C72.2-Olfactory nerve','C72.3-Optic nerve','C72.4-Acoustic nerve','C72.5-Cranial nerve, NOS','C72.8-Overlapping lesion of brain & CNS','C72.9-Nervous system, NOS','C75.1-Pituitary gland','C75.2-Craniopharyngeal duct','C75.3-Pineal gland'

AND {Site and Morphology.ICD-O-3 Hist/behav} = '9530/0: Meningioma, NOS','9530/1: Meningiomatosis, NOS','9530/3: Meningioma, malignant','9531/0: Meningothelial meningioma','9531/1: Meningiothelial meningioma, borderline','9531/3: Meningiothelial meningioma, malignant','9532/0: Fibrous meningioma','9532/3: Fibrous meningioma, malignant','9533/0: Psammomatous meningioma','9533/3: Psammomatous meningioma, malignant','9534/0: Angiomatous meningioma','9534/3: Angiomatous meningioma, malignant','9537/0: Transitional meningioma','9537/3: Transitional meningioma, malignant','9538/0: Clear cell meningioma, benign','9538/1: Clear cell meningioma','9538/3: Papillary meningioma',

Select Only: Cases in Research Database
